# Author Correction: Structure-based design of a dual-warhead covalent inhibitor of FGFR4

**DOI:** 10.1038/s42004-022-00672-w

**Published:** 2022-04-14

**Authors:** Xiaojuan Chen, Huiliang Li, Qianmeng Lin, Shuyan Dai, Sitong Yue, Lingzhi Qu, Maoyu Li, Ming Guo, Hudie Wei, Jun Li, Longying Jiang, Guangyu Xu, Yongheng Chen

**Affiliations:** 1 Department of Oncology, NHC Key Laboratory of Cancer Proteomics, State Local Joint Engineering Laboratory for Anticancer Drugs, Xiangya Hospital, Central South University, Changsha, Hunan China; 2 Key Laboratory of Chemical Biology and Traditional Chinese Medicine, Ministry of Educational of China, Key Laboratory of the Assembly and Application of Organic Functional Molecules of Hunan Province, College of Chemistry and Chemical Engineering, Hunan Normal University, Changsha, Hunan China; 3 Department of Pathology, Xiangya Hospital, Central South University, Changsha, Hunan China; 4 National Clinical Research Center for Geriatric Disorders, Xiangya Hospital, Central South University, Changsha, Hunan China

**Keywords:** X-ray crystallography, Kinases, Kinases, Drug discovery

Correction to: *Communications Chemistry* 10.1038/s42004-022-00657-9, published online 17 March 2022.

The original version of this Article contained a number of errors in Fig. 2, its caption, and the Results section. In Fig. 2, the conversion from **6** to **10** incorrectly depicted the addition of 2,3-diaminotoluene instead of 1-amino-2-methyl-6-nitrobenzene; the chemical structure of **10** incorrectly depicted an amino substituent in place of the nitro group; and the initial step in the conversion from **10** to **CXF-008**, i.e., the addition of Raney Ni and H_2_, was omitted in error. The caption of Fig. 2 and the 5^th^ sentence of the Results section incorrectly named uracil in place of 5-hydroxymethyluracil as the starting material. These errors have now been corrected in both the PDF and HTML versions of the Article.

