# Supplementary material for: Structure-based design of a dual-warhead covalent inhibitor of FGFR4
Source: Commun Chem. 2022 Mar 17;5:36. doi: 10.1038/s42004-022-00657-9 (PMC9814781; doi:10.1038/s42004-022-00657-9)
Supplement: Supplementary file 2 — Supplementary Information [file 42004_2022_657_MOESM2_ESM.pdf]

Supporting information

Structure-based design of a dual-warhead covalent inhibitor of FGFR4

Xiaojuan Chen[1,#], Huiliang Li[2,#], Qianmeng Lin[1], Shuyan Dai[1], Sitong Yue[1], Lingzhi Qu[1], Maoyu Li[1], Ming Guo[1], Hudie Wei[1], Jun Li[1], Longying Jiang[1,3,*], Guangyu Xu [2,*], Yongheng Chen [1,4,*]

[1]Department of Oncology, NHC Key Laboratory of Cancer Proteomics, State Local Joint Engineering Laboratory for Anticancer Drugs, Xiangya Hospital, Central South University, Changsha, Hunan, China

[2]Key Laboratory of Chemical Biology and Traditional Chinese Medicine, Ministry of Educational of China, Key Laboratory of the Assembly and Application of Organic Functional Molecules of Hunan Province, College of Chemistry and Chemical Engineering, Hunan Normal University, Changsha, Hunan, China

[3]Department of Pathology, Xiangya Hospital, Central South University, Changsha, Hunan, China

[4]National Clinical Research Center for Geriatric Disorders, Xiangya Hospital, Central South University, Changsha, Hunan, China

[#]These authors contribute equally.

*To whom correspondence should be addressed. Tel: +86 731 84327542; Fax: +86 731 84327542; Email: yonghenc@163.com (Y.C.)

*Correspondence may also be addressed to gyxu@hunnu.edu.cn (G.X.), longyingj1024@163.com (L.J.)

Supplementary Methods **3**

Synthetic Methods 3

Supplementary Figures **8**

Supplementary Fig. 1 8

Supplementary Fig. 2 9

Supplementary Fig. 3 10

Supplementary Fig. 4 11

Supplementary Fig. 5 12

Supplementary Fig. 6 13

Supplementary Fig. 7 14

Supplementary Fig. 8 15

Supplementary Fig. 9 16

Supplementary Tables **17**

Supplementary Table 1 17

Supplementary Table 2 18

Supplementary Methods

Synthetic Methods

Unless otherwise noted, all reagents were obtained via commercial sources and used without further purification. The ^{1}H NMR and ^{13}C NMR spectra were recorded in $CDCl_3$ and DMSO-d6 using Brücker 500 MHz NMR spectrometer; the chemical shifts are reported in δ ppm relative to TMS. The mass spectrum was recorded on a Waters XEVO LC-MS spectrometer.

*2,4-Dichloro-5-(chloromethyl)pyrimidine (**2**)*

DIPEA (14.5 g, 112.4 mmol) was added dropwise to a mixtue of 5-hydroxymethylpyrimidine-2,4-diol 2 (5.00 g, 35.2 mmol) and phosphorus oxychloride (16.5 mL, 177.5 mmol) in 10 mL toluene at 0°C. After stirring for 5 min, the reaction solution was heated to 125°C and stirred for 5h. The solution was cooled to room temperature then 50 mL ice water was added slowly to quench the reaction. The mixture was extracted with toluene (3×50 mL) and the organic phase was dried with anhydrous Na_2SO_4 then evaporated under vacuum. The residue was purified by column chromatography on silica gel to give compound 2 as a colorless oil (5.2 g, yield 74.9 %). ^{1}H NMR (500 MHz, DMSO-d6): δ 8.97 (s, 1H), 4.86 (s, 2H).

*N-((2,4-Dichloropyrimidinyl)-5-methyl)-3,5-dimethoxyaniline (**3**)*

A mixture of compound **2** (4.00 g, 20.3 mmol) and potassium iodide (3.50 g, 21.0 mmol) in 25 mL acetone was stirred 25°C for 15 min, then heated to 60°C for 30 mins. The mixture was filtered while hot, the filtrate was cooled to room temperature then 3,5-dimethoxyaniline (3.7 g, 24.2 mmol) and potassium carbonate (4.8 g, 34.5 mmol) was added. The mixture was stirred for 10 h at room temperature. After the reaction was complete, the solvent was evaporated under vacuum. The residue was added 25 mL ethanol and stirred for 30 mins under ice-cooling. The precipitant was filtered and dried under vacuum to give compound **3** as a white solid (5.3g, 84.4%). ^{1}H NMR (500 MHz, CDCl3): δ 8.51 (s, 1H), 5.92 (s, 1H), 5.72 (s, 2H), 4.39 (s, 2H), 4.27 (s, 1H), 3.72 (s, 6H).

*tert-butyl 4-(3-aminopropyl)piperazine-1-carboxylate (**4**)*

A 50 mL flask was charged with N-(3-bromopropyl)phthalimide **8** (10.00 g, 37.3 mmol), 1-boc-piperazine (7.00 g, 37.6 mmol), potassium iodide (12.40 g, 74.6 mmol), potassium carbonate (8.88 g, 63.4 mmol) and 50 mL N, N-Dimethylacetamide. The mixture was stirred for 18h at room temperature and diluted with 150 mL ethyl acetate. The salts were removed by filtration and the filtrate was washed with saturated aq. $NaHCO_3$ (30 mL) and saturated aq. NaCl (50 mL). The organic phase was dried with anhydrous Na_2SO_4 and concentrated under vacuum. The residue was slurried in 20 mL ethyl acetate. The precipitant was filtered and dried to give compound **9** as a white solid (12.2g, 87.6%). A solution of compound **9** (7.40 g, 19.8 mmol) in 8 ml hydrazine hydrate and 30 ml ethanol was stirred for 3 h at 70 °C. After the reaction was complete, the mixture was cooled to room temperature, the precipitant was filtered and the filtrate was concentrated under vacuum. The residue was added 20 mL ether and a small amount of anhydrous Na_2SO_4 then stirred for 10min under ice bath. After filtration separation, the filtrate was concentrated under vacuum to give compound **4** as a colorless oil (3.9 g, yield 81.2%), which was used in next step without further purification. ^{1}H NMR (500 MHz, CDCl3): δ 3.41 (t, J = 4.8 Hz, 4H), 2.74 (t, J = 6.8 Hz, 4H), 2.38 (q, J = 7.2 Hz, 6H), 1.62 (m, J = 7.0 Hz, 2H), 1.44 (s, 9H).

*tert-Butyl 4-(3-((2-chloro-5-(((3,5-dimethoxyphenyl)amino)methyl)pyrimidin-4-yl)amino)propyl)piperazine-1-carboxylate (**5**)*

A flask was charged with compound **3** (2.51 g, 8.0 mmol), compound **4** (2.58 g, 10.6 mmol) and DIPEA (4.2 mL, 25.4 mmol) and 20 mL dry dioxane. The mixture was heated to 60 °C and stirred for 10h. After the reaction was complete, the solvent was evaporated under vacuum. The residue was purified by column chromatography on silica gel to give compound **5** as a pale yellow oil (3.16 g, yield 76.0%). ^{1}H NMR (500 MHz, CDCl3): δ 7.89 (d, J = 1.4 Hz, 1H), 6.55 (s, 1H), 5.97 (t, J = 2.0 Hz, 1H), 5.86 (d, J = 2.1 Hz, 2H), 4.07 (d, J = 3.3 Hz, 2H), 3.74 (s, 6H), 3.73 (s, 2H), 3.55 (q, J = 6.1 Hz, 2H), 3.37 (s, 4H), 2.35 (s, 4H), 1.45 (s, 2H), 1.44 (s, 9H).

*tert-Butyl 4-(3-(7-chloro-3-(3,5-dimethoxyphenyl)-2-oxo-3,4-dihydropyrimidinyl[4,5-d]pyrimidin-1(2H)-yl)propyl)-1-carboxylate (**6**)*

Triethylamine (0.80 mL, 5.76 mmol) was added dropwise into a solution of compound

5 (1.50 g, 2.88 mmol), triphosgene (427 mg, 1.44 mmol) and 15 mL dry THF under ice cool. The reaction solution was stirred for 1 h at 0 °C then heated to reflux for 10 h. After the reaction was complete, the mixture was cooled to 0 °C and 5 mL of ice water was added to quench the reaction. The mixture was concentrated in vacuo then extracted with ethyl acetate (3×20 mL). The organic phase was washed with saturated sodium bicarbonate (10 mL) and water (20 mL) then dried over anhydrous Na2SO4. After the solvent was evaporated under reduced pressure, the resulting yellow residue was purified by flash chromatography on silica gel to give 1.36 g of crude product, which was recrystallized with isopropanol/petroleum ether to give pure compound **6** as a white solid (920mg, 58.4 % yield). ^{1}H NMR (500 MHz, CDCl3): δ 8.12 (s, 1H), 6.45 (d, J = 2.15 Hz, 2H), 6.41 (t, J = 2.15 Hz, 1H), 4.74 (s 2H), 4.15 (t, J = 7.25 Hz, 2H), 3.79 (s, 6H), 3.51 (s, 4H), 2.58 (s, 4H), 2.52 (s, 2H), 1.99 (s, 2H), 1.45 (s, 9H).

*tert-Butyl 4-(3-(3-(3,5-dimethoxyphenyl)-7-((2-methyl-6-nitrophenyl)amino)-2-oxo-3,4-dihydropyrimido[4,5-d]pyrimidin-1(2H)-yl)propyl)piperazine-1-carboxylate **(10)***

To a 25 mL Schlenk tube was added compound **6** (850 mg, 1.55 mmol), 2-methyl-6-nitrophenylamine (354 mg, 2.33 mmol), Cs_2CO_3 (1.51 g, 4.65 mmol), X-Phos (151 mg, 0.31 mmol), $Pd_2(dba)_3$ (146 mg, 0.16 mmol) and 3 mL dry DMA under nitrogen atomosphere, the mixture was heated to 110 °C and stirred for 3h. The reaction solution was then allowed to cool to room temperature, diluted with 50 mL water and 90 mL ethyl acetate. The organic phase was washed with saturated aq. $NaHCO_3$ and dried with anhydrous Na_2SO_4. The solvent was evaporated in vacuo and the crude material was purified by flash chromatography on silica gel to give 620 mg of compound **10** mixed with a small mount of des-Boc product, which was used in the next reaction. MS $[M+H]^+$ m/z calculated for $C_{33}H_{43}N_8O_7$, 663.3; found, 663.3.

*N-(2-((8-(3-(4-Acryloylpiperazin-1-yl)propyl)-6-(3,5-dimethoxyphenyl)-7-oxo-5,6,7,8-tetrahydropyrimido[4,5-d]pyrimidin-2-yl)amino)-3-methylphenyl)acrylamide **(CXF-008)***

After three vacuum-hydrogen exchanges a mixture of compound **10** (600 mg, 0.91 mmol) and Raney nickel (W-2, 1.7ml) in 20 mL methanol was stirred for 10h at ambient temperature. The reaction mixture was filtered using a celite cake and the filtrate was

concentrated in vacuo. The above hydrogenated product was mixed with 1 mL trifluoroacetic acid and 15 mL dry dichloromethane, the mixture was stirred for overnight at room temperture. After the reaction was complete, the mixture was added 30 mL saturated sodium bicarbonate solution under ice bath and stirred for 10 min. The solution was extracted with dichloromethane (3×20 mL) and washed with saturated sodium bicarbonate. The organic phase was dried with anhydrous Na_2SO_4 then concentrated in vacuo to give a yellow oil, which was mixed with triethylamine (211.5 mg, 1.94 mmol) and 22 mL dry dichloromethane, then a solution of acryloyl chloride (157.5 mg, 1.76 mmol) in 1 mL dichloromethane was added dropwise. The mixture was stirred 30min at room temperature then 4.5 mL ice water was added to quench the reaction. The mixture was extracted with dichloromethane (30 mL×3) and the organic phase was washed with saturated aq. $NaHCO_3$ (20 mL) and NaCl (40 mL). After drying over anhydrous Na_2SO_4, the solvent was evaporated under reduced pressure. The residue was purified by column chromatography on silica gel to give **CXF-008** as a white powder (79.2 mg, yield 14.4%). ^1H NMR (500 MHz, $CDCl_3$): δ 8.32 (s, 1H), 7.93 (s, 1H), 7.91 (s, 1H), 7.21 (t, J = 7.8 Hz, 1H), 7.07 (d, J = 7.1 Hz, 1H), 6.88 (s, 1H), 6.54 (q, J = 10.6 Hz, 1H), 6.44 (d, J = 2.1 Hz, 2H), 6.37 (m, J = 2.75 Hz, 2H), 6.28 (t, J = 1.7 Hz, 1H), 6.20 (q, J = 10.4 Hz, 1H), 5.69 (m, J = 8.15 Hz, 2H), 4.61 (s, 2H), 3.86 (s, 2H), 3.77 (s, 6H), 3.63 (s, 2H), 3.51 (s, 2H), 2.34 (s, 4H), 2.25 (s, 3H), 1.72 (s, 3H); ^{13}C NMR (500 MHz, $CDCl_3$): δ 165.46, 161.36, 161.23, 161.02, 157.55, 153.14, 152.85, 143.88, 136.23, 131.48, 128.00, 127.73, 127.55, 127.38, 126.91, 104.22, 102.83, 99.13, 55.60, 53.05, 52.60, 47.48, 45.76, 41.94, 40.12, 24.77, 18.74; HRMS $[M+H]^+$ m/z calculated for $C_{34}H_{41}N_8O_5$, 641.3200; found, 641.3198.

tert-butyl 4-(3-(7-((2-aminophenyl)amino)-3-(3,5-dimethoxyphenyl)-2-oxo-3,4-dihydropyrimido[4,5-d]pyrimidin-1(2H)-yl)propyl)piperazine-1-carboxylate (7)

To a 25 mL Schlenk tube was added compound **6** (547 mg, 1.0 mmol), 1,2-diaminobenzene (162 mg, 1.5 mmol), Cs_2CO_3 (975 mg, 3.0 mmol), X-Phos (98 mg, 0.2 mmol), Pd2(dba)3 (92 mg, 0.1 mmol) and 3 mL dry DMA under nitrogen atomosphere, the mixture was heated to 110 °C and stirred for 3h. The reaction solution was then allowed to cool to room temperature, diluted with 50 mL water and 90 mL ethyl acetate.

The organic phase was washed with saturated aq. $NaHCO_3$ and dried with anhydrous Na_2SO_4. The solvent was evaporated in vacuo and the crude material was purified by flash chromatography on silica gel to give 328 mg (53.1%) of compound **7** as a yellow oil. ^{1}H NMR (500 MHz, CDCl3): δ 7.93 (d, J = 7.1 Hz, 1H), 7.03 (d, J = 6.0 Hz, 2H), 6.80 (s, 2H), 6.45 (s, 2H), 6.37 (s, 1H), 4.59 (s, 2H), 3.97 (s, 2H), 3.85 (s, 2H), 3.77 (s, 6H), 3.38 (s, 1H), 2.32 (s, 6H), 1.84 (s, 2H), 1.44 (s, 9H). 13C NMR (500 MHz, CDCl3): δ 161.07, 160.677, 157.15, 154.77, 153.16, 152.96, 143.94, 141.37, 126.48, 125.83, 125.68, 119.14, 116.98, 104.05, 102.27, 99.01, 79.60, 55.99, 55.50, 52.84, 47.41, 40.07, 28.45, 25.06.

N-(2-((8-(3-(4-Acryloylpiperazin-1-yl)propyl)-6-(3,5-dimethoxyphenyl)-7-oxo-5,6,7,8-tetrahydropyrimidinyl[4,5-d]pyrimidin-2-yl)amino)phenyl)acrylamide ***(CXF-009)***

A mixture of compound **7** (320 mg, 0.52 mmol) and 1 mL trifluoroacetic acid in 15 mL dry dichloromethane was stirred for overnight at room temperature. After the reaction was complete, the mixture was added 30 mL saturated sodium bicarbonate solution under ice bath and stirred for 10 min. The solution was extracted with dichloromethane (3×20 mL) and washed with saturated sodium bicarbonate. The organic phase was dried with anhydrous Na_2SO_4 then concentrated in vacuo to give a yellow oil, which was mixed with triethylamine (94 mg, 0.86 mmol) and 10 mL dry dichloromethane, then a solution of acryloyl chloride (70 mg, 0.78 mmol) in 1 mL dichloromethane was added dropwise. The mixture was stirred 30min at room temperature then 2 mL ice water was added to quench the reaction. The mixture was extracted with dichloromethane (20 mL×3) and the organic phase was washed with saturated aq. NaHCO3 (10 mL) and NaCl (20 mL). After drying over anhydrous Na2SO4, the solvent was evaporated under reduced pressure. The residue was purified by column chromatography on silica gel to give **CXF-009** as a white powder (36mg, yield 14.7%). ^{1}H NMR (500 MHz, CDCl3): δ 8.62 (s, 1H), 7.92 (s, 1H), 7.63 (q, J = 8.4 Hz, 3H), 7.16 (t, J = 8.2 Hz, 3H), 6.53 (q, J = 5.9 Hz, 1H), 6.45 (s, 2H), 6.39 (t, J = 12.2 Hz, 2H), 6.25 (t, J = 9.7 Hz, 2H), 5.69 (q, J = 7.3 Hz, 2H), 4.60 (s, 2H), 3.97 (s, 2H), 3.76 (s, 6H), 3.58 (s, 2H), 3.47 (s, 2H), 2.34 (s, 4H), 2.30 (s, 2H), 1.81 (s, 2H). HRMS $[M+H]^+$ m/z calculated for C33H39N8O5, 627.3043; found, 627.3036.

Supplementary Figures

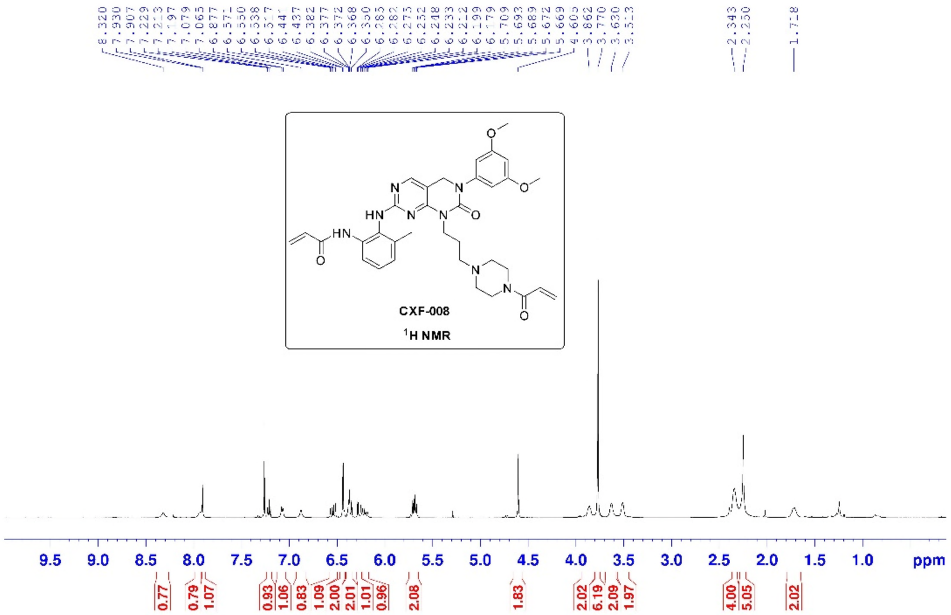

Supplementary Fig. 1. ^{1}H NMR of **CXF-008**.

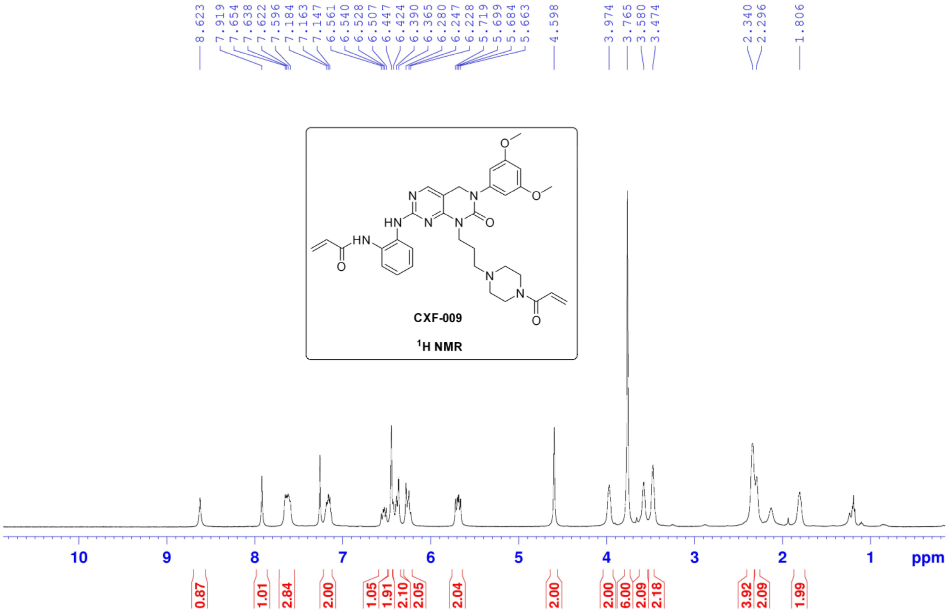

Supplementary Fig. 2. ^{1}H NMR of **CXF-009**.

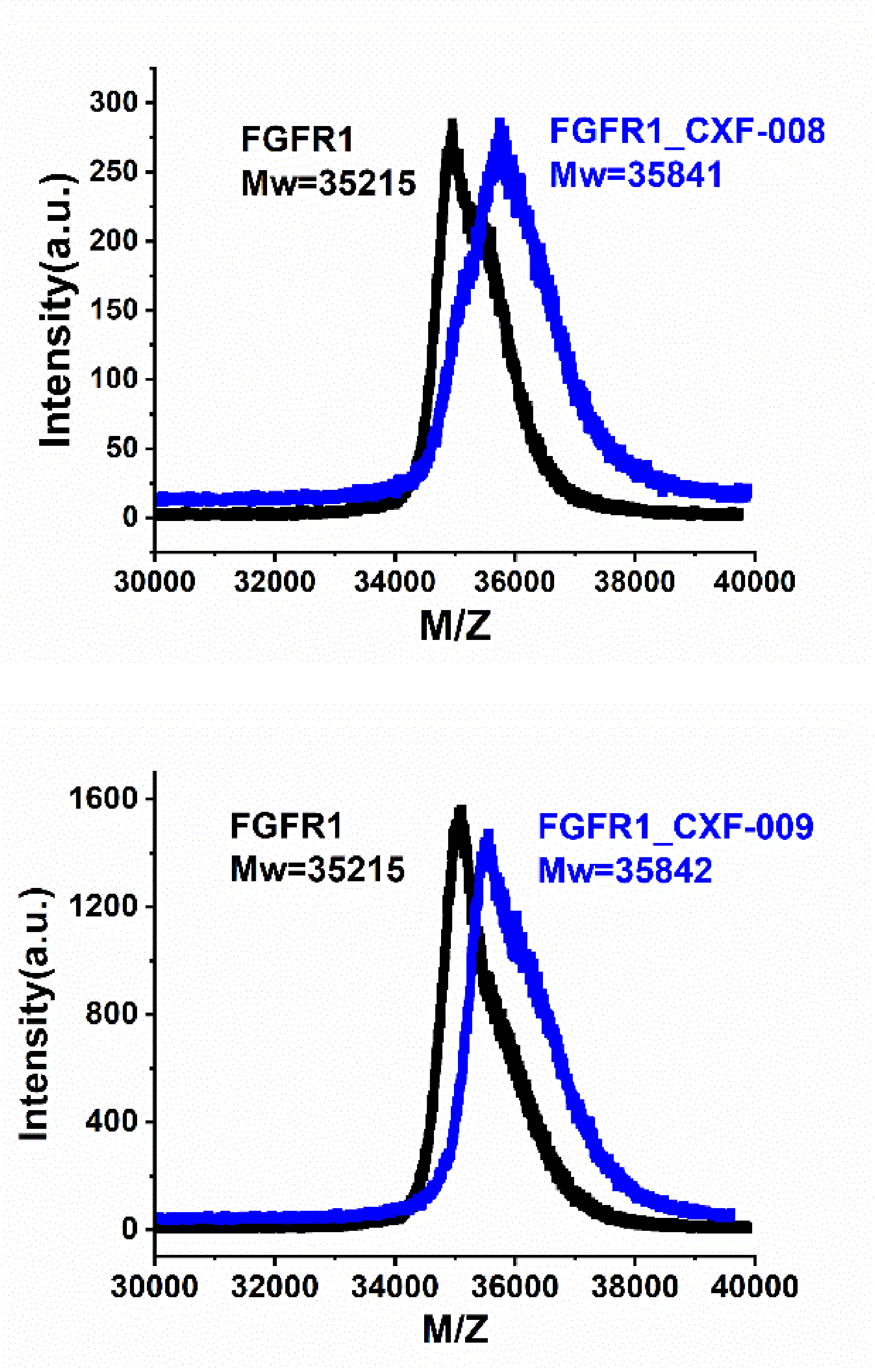

Supplementary Fig. 3. MALDI-TOF MS determination of FGFR1 (black) and FGFR1/inhibitor complexes (blue).

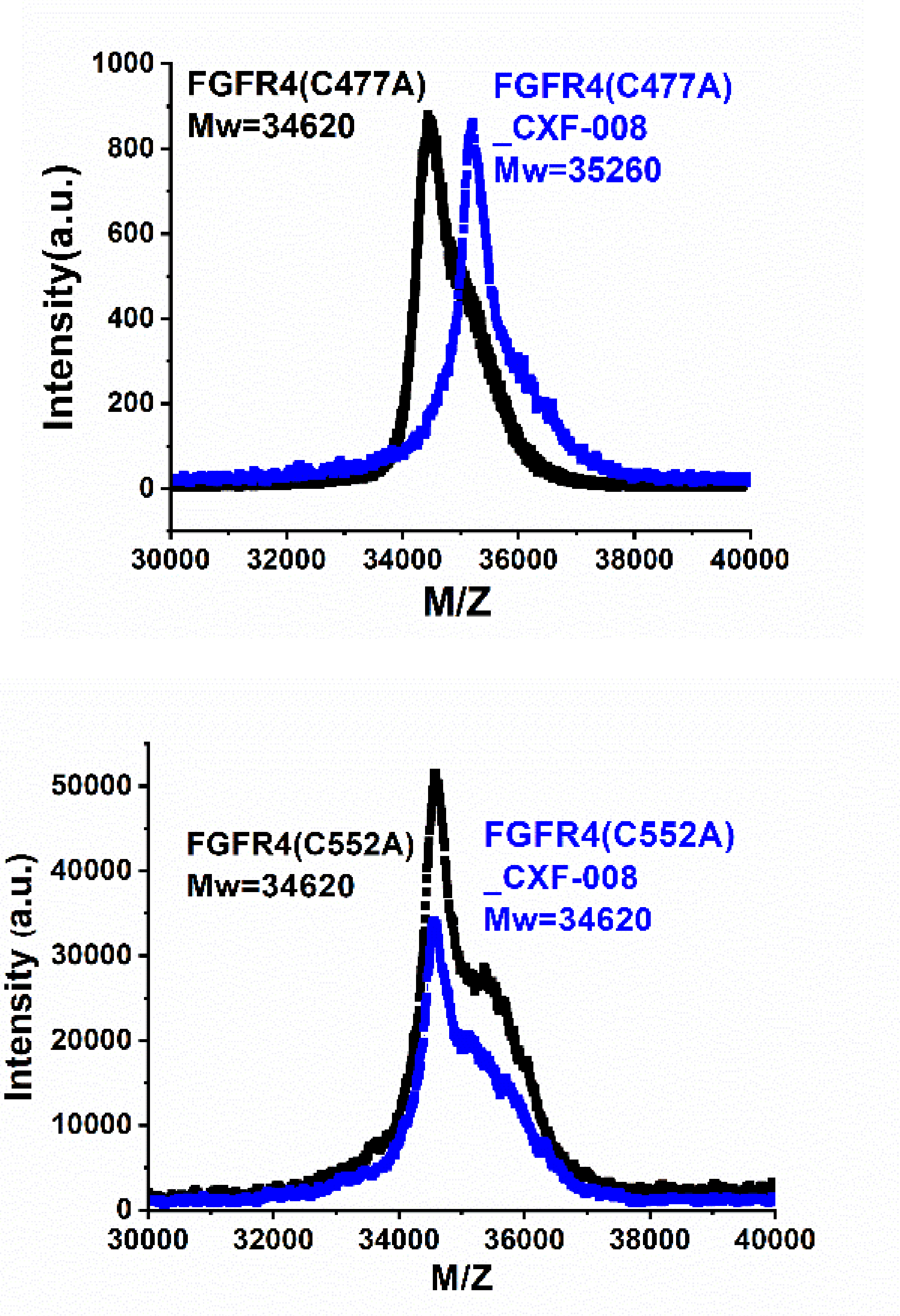

Supplementary Fig. 4. MALDI-TOF MS determination of protein (black) and protein/inhibitor complexes (blue) for **CXF-008**.

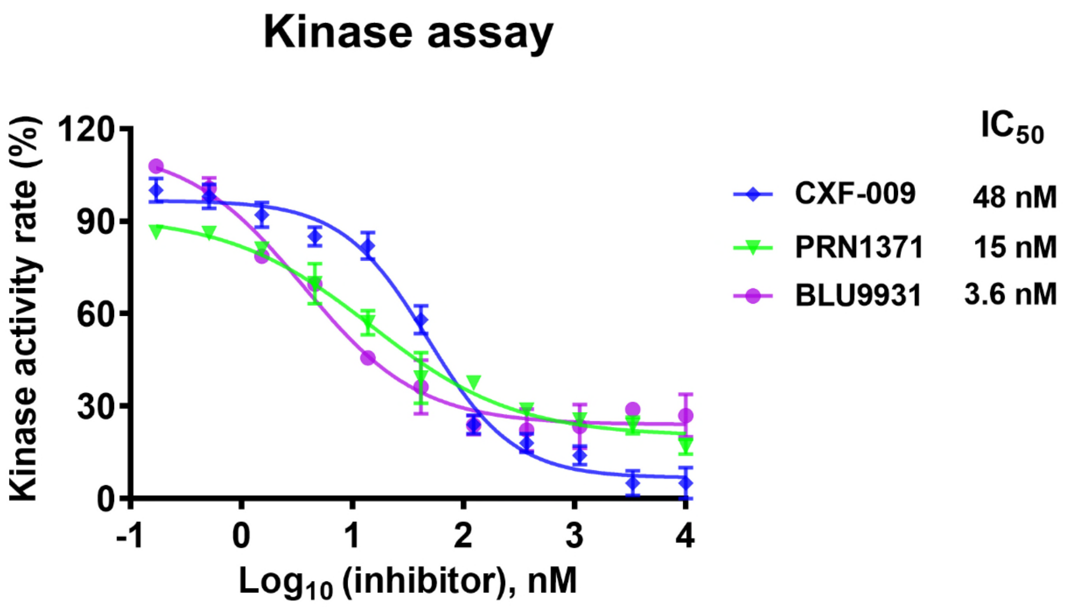

Supplementary Fig. 5. The inhibitory activities of **CXF-009** and positive control against FGFR4 using kinase assay. Data are representative of 3 independent replicates. Error bars indicate mean ± SD.

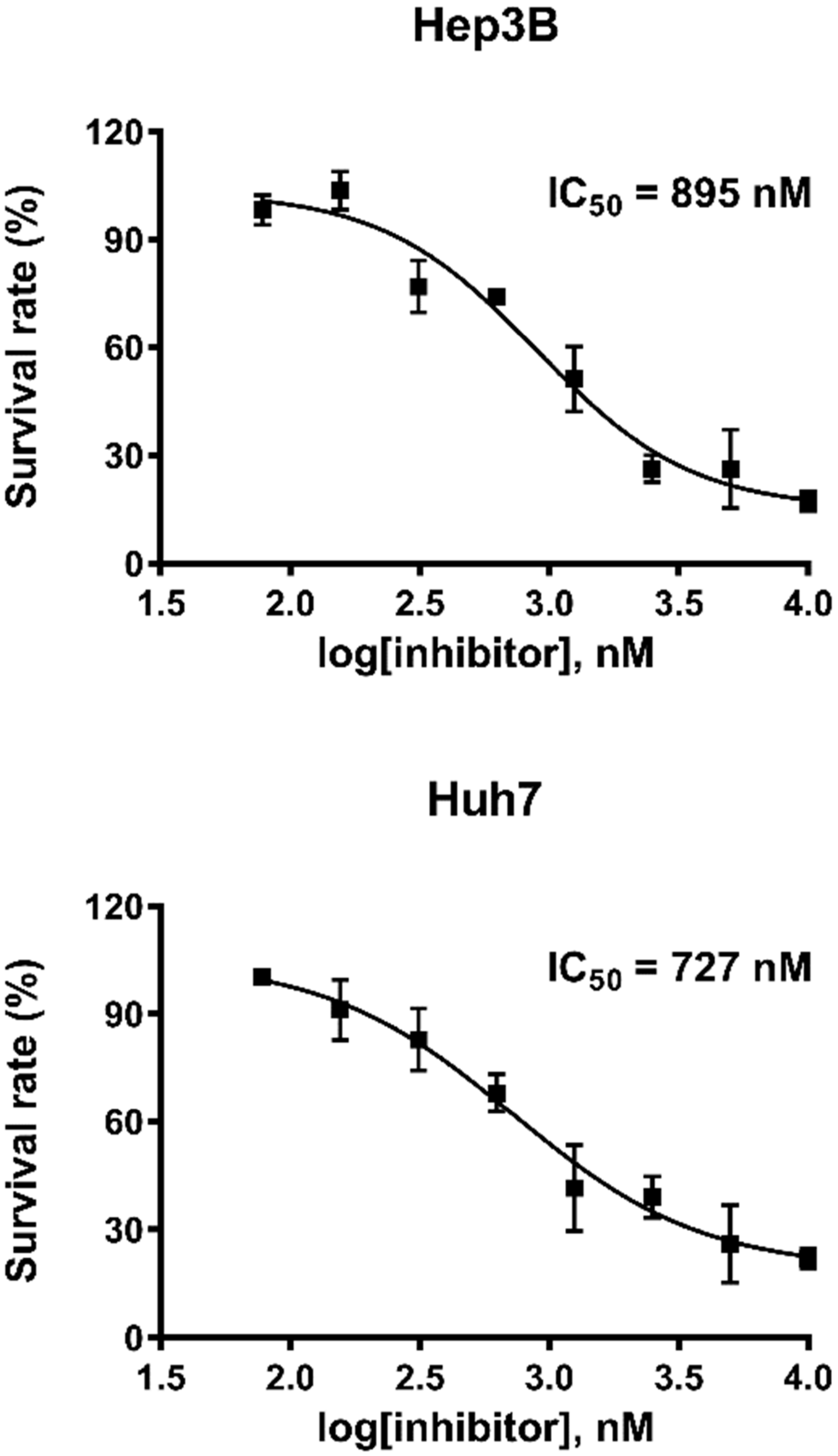

Supplementary Fig. 6. The inhibitory activities of **CXF-009** for Hep3B and Huh7 cells. Data are representative of 3 independent replicates. Error bars indicate mean ± SD.

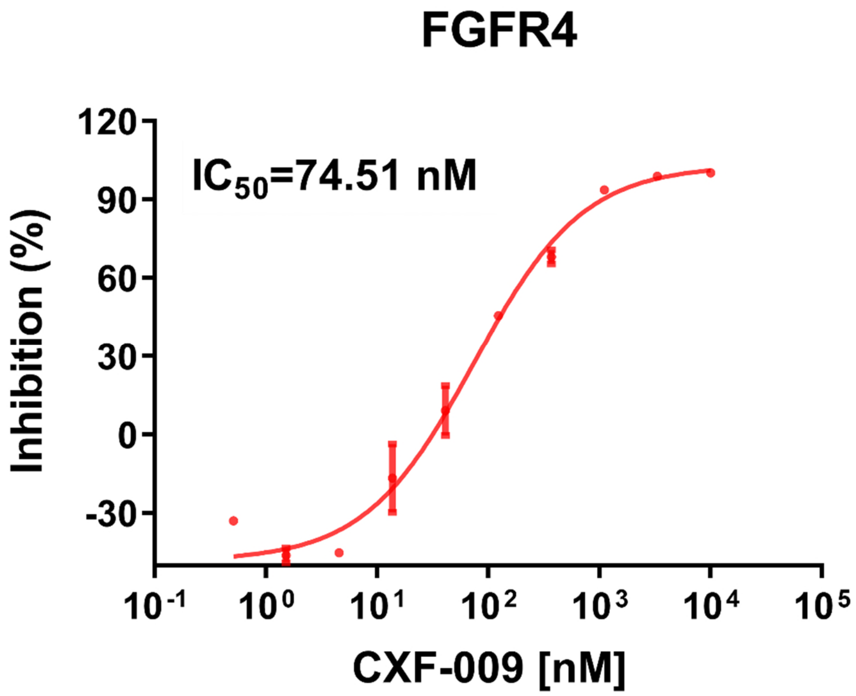

Supplementary Fig. 7. The IC_{50} of **CXF-009** against FGFR4 using HTRF assay. Data are presented as dot, n = 3 independent replicates. Error bars indicate mean ± SD.

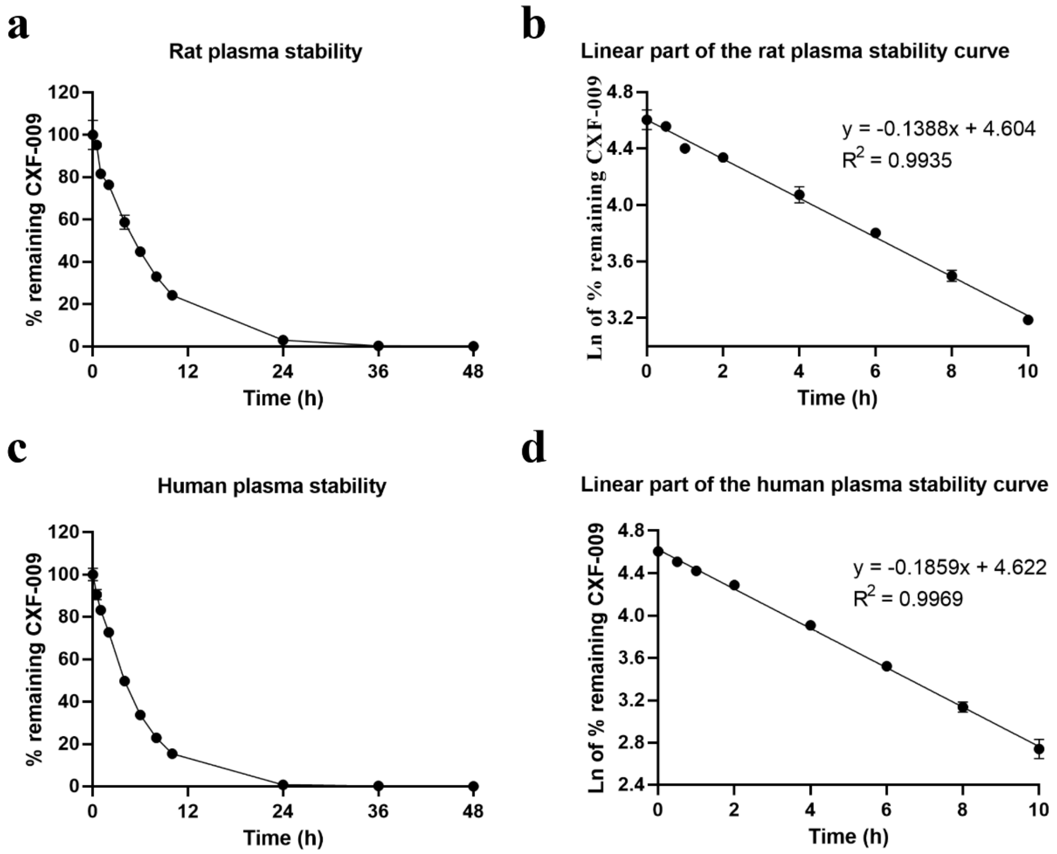

Supplementary Fig. 8. The plasma stability of **CXF-009**.The plasma stability curve of **CXF-009** in rat plasma (a) and human plasma (c). The corresponding linear regression of the linear part of the plasma stability curve (b/d)

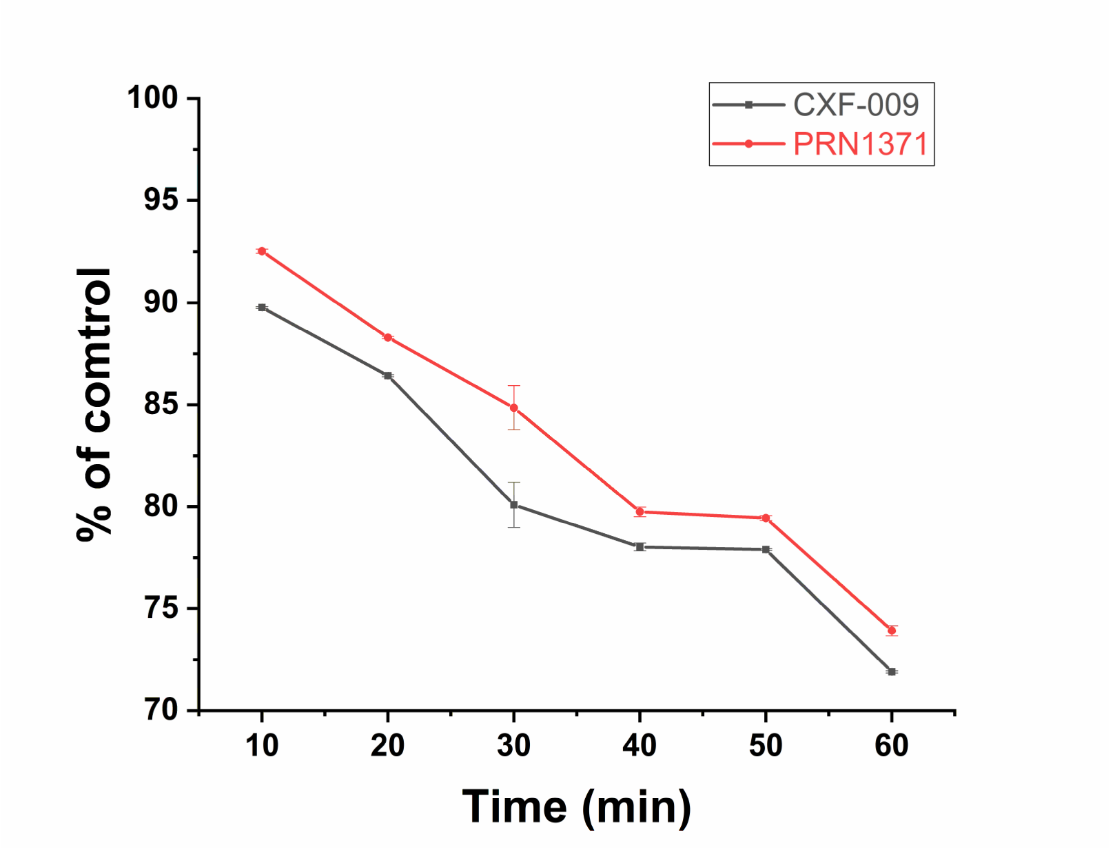

Supplementary Fig. 9. The reaction of GSH with **CXF-009** and PRN1371. Data are presented as dot, n = 3 independent replicates. Error bars indicate mean ± SD.

Supplementary Tables

Supplementary Table 1. Data collection and refinement statistics (molecular replacement)

	FGFR4/CXF-009
**Data collection**	
Space group	P 1 21 1
Cell dimensions	
a, b, c (Å)	69.42, 61.31, 76.53
α, β, γ (°)	90.00, 112.05, 90.00
Resolution (Å)	64.34 - 1.983 (2.053 - 1.983)
R_{sym} or R_{merge}	0.13/0.12 (0.33)
I / σI	10.96 (2.37)
Completeness (%)	99.81 (98.47)
Redundancy	6.7 (6.5)
**Refinement**	
Resolution (Å)	64.34 - 1.983
No. reflections	276118 (26441)
R_{work} / R_{free}	0.19/0.24
No. atoms	
Protein	583
Ligand/ion	117
Water	421
B-factors	27.90
Protein	27.00
Ligand/ion	36.00
Water	34.50
R.m.s. deviations	
Bond lengths (Å)	0.007
Bond angles (°)	1.05

Supplementary Table 2. The list of the tested kinases and their %inhibition at 1000 nM of **CXF-009**

number	Kinases	%inhibition	number	Kinases	%inhibition
1	FGFR4	94.15	42	KHS	4.11
2	TRKB	42.11	43	Erk1	3.99
3	FER	37.10	44	AKT1	3.98
4	TSSK1	30.01	45	MER	3.59
5	TRKA	26.25	46	SRC	3.48
6	PIM1	24.24	47	AKT2	3.35
7	IKK-beta	18.96	48	CSK	3.29
8	DCAMKL2	18.89	49	PAK1	3.22
9	NEK2	17.87	50	PAK2	3.22
10	PIM3	17.54	51	SGK	2.90
11	MINK	16.57	52	AurC	2.77
12	FMS (CSF1R)	15.43	53	EPHA7	2.74
13	AurA	15.38	54	GLK	2.68
14	PI3K(p120gamma)	15.01	55	MET	2.65
15	Erk5	14.01	56	CK1α(CSNK1A1)	2.60
16	p38δ	13.91	57	HGK	2.58
17	SYK	13.61	58	DAPK1	2.52
18	DCAMKL1	13.45	59	PIM2	2.38
19	ZAP70	11.85	60	IKK-alpha(CHUK)	2.35
20	FGFR3	11.07	61	HIPK4	2.32
21	HER2	10.66	62	p38β	2.25
22	p38α	10.55	63	RSK2	2.24
23	BMX	10.46	64	AMPKα1/β1/γ1	2.23
24	EPHA5	8.67	65	EPHA1	2.15
25	TRKC(NTRK3)	8.57	66	DDR2	2.15
26	LYNb	7.62	67	EPHA2	2.12
27	RSK3	7.58	68	PYK2	2.11
28	ALK	7.28	69	JAK3	1.94
29	IKKε(IKBKE)	7.21	70	BRK	1.91
30	TTK	6.62	71	DYRK4	1.84
31	PAK4	6.50	72	Pkca	1.73
32	FLT1 (VEGFR1)	5.91	73	RET	1.72
33	GSK3α	5.79	74	p70S6K	1.69
34	SRM	5.29	75	ABL2	1.61
35	EPHA6	5.28	76	CDK2/CycE1	1.59
36	NEK9	5.11	77	SGK3	1.58
37	ROCK1	5.09	78	CHK1(CHEK1)	1.53
38	FGFR1	4.98	79	CLK1	1.48
39	CK1γ1	4.78	80	MAP4K2	1.43
40	ITK	4.35	81	NEK1	1.39
41	ACK	4.18	82	CDK7/CycH/MAT1	1.27

number	Kinases	%inhibition	number	Kinases	%inhibition
83	TGFbR1	1.08	125	EPHB1	-1.49
84	PAK3	0.97	126	MAP3K3	-1.62
85	Erk2	0.94	127	BRAF[V600E]	-1.68
86	TYRO3	0.80	128	EPHA3	-1.81
87	INSR	0.80	129	EPHB4	-1.86
88	FLT4 (VEGFR3)	0.74	130	IGF1R	-2.13
89	Her4(ERBB4)	0.69	131	HPK1	-2.36
90	DYRK1B	0.62	132	AurB	-2.47
91	MAP2K1	0.59	133	FYNb	-2.49
92	PLK1	0.59	134	RSK1	-2.52
93	JAK2	0.56	135	LYNa	-2.53
94	SGK2	0.51	136	ROCK2	-2.53
95	JNK2	0.40	137	CDK2/CycA2	-2.58
96	DYRK2	0.38	138	CHK2	-3.02
97	NPM1-ALK	0.37	139	EPHB2	-3.12
98	FGFR2	0.27	140	CK2α1/β	-3.22
99	ALK4	0.23	141	DDR1	-3.41
100	PIK3CB/PIK3R1	0.17	142	MUSK	-3.46
101	MAPKAPK2	0.15	143	LCK	-3.46
102	p38γ	0.13	144	IRR	-3.50
103	CDK9/CycK	0.11	145	JNK3	-3.67
104	PKD2	0.08	146	CDK18/CycY	-3.74
105	RSK4	0.00	147	TAOK2	-3.76
106	FRK	-0.22	148	JNK1	-4.40
107	GSK3β	-0.37	149	RON	-4.53
108	FAK	-0.41	150	HIPK3	-4.60
109	ROS1	-0.51	151	CK1ε	-4.60
110	AKT3	-0.52	152	CK1γ2	-4.80
111	EPHA8	-0.52	153	BRSK2	-5.03
112	CLK3	-0.52	154	HIPK2	-5.04
113	TAOK1	-0.59	155	TYK2	-5.05
114	EPHB3	-0.82	156	KDR (VEGFR2)	-5.37
115	CDK5/p35NCK	-0.87	157	CDK5/p25NCK	-5.50
116	PDGFRa	-0.91	158	HIPK1	-5.67
117	BRAF	-0.93	159	BTK	-5.76
118	CDK1/CycA2	-1.02	160	KIT[D816V]	-6.19
119	FGR	-1.10	161	EPHA4	-6.83
120	FLT3	-1.11	162	PDGFRβ	-6.88
121	PLK3	-1.12	163	CDK3/CycE1	-7.17
122	DYRK1A	-1.23	164	CDK1/CycE1	-7.18
123	CK1δ	-1.29	165	AXL	-7.53
124	ABL1	-1.44	166	YES	-7.61

number	Kinases	%inhibition
167	FES	-7.81
168	CK1γ3	-7.86
169	CDK16/CycY	-7.90
170	CK2α2/β	-7.98
171	MST2	-8.12
172	YSK1	-8.51
173	FYNa	-9.44
174	CDK6/CycD1	-9.58
175	TGFβR2	-10.45
176	HCK	-11.64
177	DYRK3	-12.01
178	JAK1	-12.31
179	TIE2	-12.75
180	TNIK	-13.41
181	KIT	-14.35
182	EGFR	-16.29
183	PLK2	-19.48
184	PIK3CA/PIK3R1	-34.97
185	PIK3CD/PIK3R1	-44.43
