# Supplementary material for: Structure-based design of a dual-warhead covalent inhibitor of FGFR4
Source: Commun Chem. 2022 Mar 17;5:36. doi: 10.1038/s42004-022-00657-9 (PMC9814781; doi:10.1038/s42004-022-00657-9)
Supplement: Supplementary file 3 — Reporting Summary [file 42004_2022_657_MOESM3_ESM.pdf]

nature research

Corresponding author(s): Yongheng Chen, Guangyu Xu, Longying Jiang

Last updated by author(s): Feb 14, 2022

Reporting Summary

Nature Research wishes to improve the reproducibility of the work that we publish. This form provides structure for consistency and transparency in reporting. For further information on Nature Research policies, see our Editorial Policies and the Editorial Policy Checklist.

Statistics

For all statistical analyses, confirm that the following items are present in the figure legend, table legend, main text, or Methods section.

n/a	Confirmed	
☐	☒	The exact sample size (*n*) for each experimental group/condition, given as a discrete number and unit of measurement
☐	☒	A statement on whether measurements were taken from distinct samples or whether the same sample was measured repeatedly
☒	☐	The statistical test(s) used AND whether they are one- or two-sided<br>*Only common tests should be described solely by name; describe more complex techniques in the Methods section.*
☒	☐	A description of all covariates tested
☒	☐	A description of any assumptions or corrections, such as tests of normality and adjustment for multiple comparisons
☐	☒	A full description of the statistical parameters including central tendency (e.g. means) or other basic estimates (e.g. regression coefficient) AND variation (e.g. standard deviation) or associated estimates of uncertainty (e.g. confidence intervals)
☒	☐	For null hypothesis testing, the test statistic (e.g. *F*, *t*, *r*) with confidence intervals, effect sizes, degrees of freedom and *P* value noted<br>*Give P values as exact values whenever suitable.*
☒	☐	For Bayesian analysis, information on the choice of priors and Markov chain Monte Carlo settings
☒	☐	For hierarchical and complex designs, identification of the appropriate level for tests and full reporting of outcomes
☒	☐	Estimates of effect sizes (e.g. Cohen's *d*, Pearson's *r*), indicating how they were calculated

Our web collection on statistics for biologists contains articles on many of the points above.

Software and code

Policy information about availability of computer code

Data collection	ProteinPilotTM (MALDI-TOF-MS), PerkinElmer Envision Manager (version 1.13, kinase assay, GSH Reaction Assay), UPLC-MS/MS(In Vitro Plasma Stability Study)
Data analysis	Data Explorer (TM) Software (MALDI-TOF-MS data analysis), OriginPro (version 8.0, MALDI-TOF-MS data graphics,GSH Reaction Assay), HKL2000 (version 716.1, x-ray data processing), PHENIX (version 1.10.1-2155, structure determination and refinement), WinCoot (version 0.8.9, atomic model building), GraphPad Prism 7.0 (kinase assay, Ba/F3 cells, In Vitro Plasma Stability Study and HTRF assay data analysis), PyMOL (version 2.3.3, molecular graphics and analyses)

For manuscripts utilizing custom algorithms or software that are central to the research but not yet described in published literature, software must be made available to editors and reviewers. We strongly encourage code deposition in a community repository (e.g. GitHub). See the Nature Research guidelines for submitting code & software for further information.

Data

Policy information about availability of data

All manuscripts must include a data availability statement. This statement should provide the following information, where applicable:
- Accession codes, unique identifiers, or web links for publicly available datasets
- A list of figures that have associated raw data
- A description of any restrictions on data availability

The structure model used in the study from the Protein Data Bank with accession codes: 4QRC [https://www.rcsb.org/structure/4QRC. The coordinates and structure factors are deposited in the Protein Data Bank with the accession codes 7V29. All other relevant data supporting the key findings of this study are available within the article and its Supplementary Information files. A reporting summary is available as a Supplementary Information file.

Field-specific reporting

Please select the one below that is the best fit for your research. If you are not sure, read the appropriate sections before making your selection.

☒ Life sciences ☐ Behavioural & social sciences ☐ Ecological, evolutionary & environmental sciences

For a reference copy of the document with all sections, see nature.com/documents/nr-reporting-summary-flat.pdf

Life sciences study design

All studies must disclose on these points even when the disclosure is negative.

Sample size	The sample size was determined based on standards in the field, attempting to have a minimum of N = 3 biological replicates with sufficient reproducibility.
Data exclusions	No data were exculded.
Replication	Biochemical was repeated three times independently with similar results. All replication were successful. Several datasets were collected from different crystals but only the datasets with highest resolution were solved and refined.
Randomization	Samples were allocated randomly.
Blinding	Blinding was not used in this study. Data were derived from instrument-based measurement and software-based analysis.

Reporting for specific materials, systems and methods

We require information from authors about some types of materials, experimental systems and methods used in many studies. Here, indicate whether each material, system or method listed is relevant to your study. If you are not sure if a list item applies to your research, read the appropriate section before selecting a response.

Materials & experimental systems

n/a	Involved in the study	
☒	☐	Antibodies
☐	☒	Eukaryotic cell lines
☒	☐	Palaeontology and archaeology
☒	☐	Animals and other organisms
☒	☐	Human research participants
☒	☐	Clinical data
☒	☐	Dual use research of concern

Methods

n/a	Involved in the study	
☒	☐	ChIP-seq
☒	☐	Flow cytometry
☒	☐	MRI-based neuroimaging

Eukaryotic cell lines

Policy information about cell lines

Cell line source(s)	Mouse ba/f3 Cells, human Hep3B and Huh7 cells was purchased from Thermofisher.
Authentication	The cell line used was not authenticated.
Mycoplasma contamination	The cells were tested negative for mycoplasma.
Commonly misidentified lines (See ICLAC register)	None were used.
